# Integrative epigenomic analysis in differentiated human primary bronchial epithelial cells exposed to cigarette smoke

**DOI:** 10.1038/s41598-018-30781-3

**Published:** 2018-08-24

**Authors:** Kimberly Glass, Derek Thibault, Feng Guo, Jennifer A. Mitchel, Betty Pham, Weiliang Qiu, Yan Li, Zhiqiang Jiang, Peter J. Castaldi, Edwin K. Silverman, Benjamin Raby, Jin-Ah Park, Guo-Cheng Yuan, Xiaobo Zhou

**Affiliations:** 1 Channing Division of Network Medicine, Brigham and Women’s Hospital and Harvard Medical School, Boston, United States; 2 Department of Enviromental Health, Harvard T.H. School of Public Health, Boston, United States; 3 Division of Pulmonary and Critical Care Medicine, Department of Medicine, Brigham and Women’s Hospital and Harvard Medical School, Boston, United States; 4 Department of Biostatistics and Computational Biology, Dana-Farber Cancer Institute, Boston, United States; 5 Department of Biostatistics, Harvard T.H. School of Public Health, Boston, United States; 6 Division of General Internal Medicine and Primary Care, Brigham and Women’s Hospital, Boston, Massachusetts USA

## Abstract

Cigarette smoke (CS) is one of the major risk factors for many pulmonary diseases, including chronic obstructive pulmonary disease (COPD) and lung cancer. The first line of defense for CS exposure is the bronchial epithelial cells. Elucidation of the epigenetic changes during CS exposure is key to gaining a mechanistic understanding into how mature and differentiated bronchial epithelial cells respond to CS. Therefore, we performed epigenomic profiling in conjunction with transcriptional profiling in well-differentiated human bronchial epithelial (HBE) cells cultured in air-liquid interface (ALI) exposed to the vapor phase of CS. The genome-wide enrichment of histone 3 lysine 27 acetylation was detected by chromatin immunoprecipitation followed by next generation sequencing (ChIP-Seq) in HBE cells and suggested the plausible binding of specific transcription factors related to CS exposure. Additionally, interrogation of ChIP-Seq data with gene expression profiling of HBE cells after CS exposure for different durations (3 hours, 2 days, 4 days) suggested that earlier epigenetic changes (3 hours after CS exposure) may be associated with later gene expression changes induced by CS exposure (4 days). The integration of epigenetics and gene expression data revealed signaling pathways related to CS-induced epigenetic changes in HBE cells that may identify novel regulatory pathways related to CS-induced COPD.

## Introduction

Among the vast number of environmental exposures, cigarette smoke (CS) exposure remains one of the greatest avoidable environmental health risks. It is estimated that ~20% of all deaths in the U.S. annually are attributable to cigarette smoke^1^, and CS exposure is the strongest modifiable risk factor for the four leading causes of death in the US: cancer, cardiovascular disease, stroke, and chronic obstructive pulmonary disease (COPD)^2^. Therefore, defining the molecular consequences of CS exposure is of great clinical and public health importance.

Elucidation of histone tail modifications has provided mechanistic insights into gene regulation for many complex diseases. One of the most widely studied histone marks of active gene transcription is acetylation of lysine 27 residues on the N-terminal tail of histone H3 (H3K27Ac), which marks the location of active enhancers or promoters^3^. For CS-related pulmonary diseases, the bronchial epithelium (BE) lining the respiratory tract is directly exposed to CS and serves as the primary defensive barrier at injured sites. Inhaled toxicants from CS lead to reversible^4^ and irreversible^5^ changes in the epigenetic landscape, including histone modification in the bronchial epithelium (BE). Indirect evidence of CS-induced airway histone modification comes from the demonstration of reduced HDAC2 activity in alveolar macrophages from smokers^6^. More direct evidence from *in vitro* studies of normal human bronchial epithelial cells has demonstrated dose- and time-dependent reduced levels of H4K16Ac and H4K20Me3, and increased levels of H3K27Me3, following exposure to CS extract^7^. However, two major gaps remain: (1) lack of direct evidence for histone modification at a genome-wide scale after CS exposure; (2) absence of data in human bronchial epithelium exposed to the vapor phase of cigarette smoke. To date, only limited epigenomic data has been generated in the respiratory tract, and very sparse genome-wide studies of the epigenomic consequences of CS have been performed^8^. Without genome-wide data, the specific mechanisms by which CS impacts normal HBE cellular function remains speculative. Given the important role of the epigenome as a biological intermediary of the consequences of CS exposure, we aimed to elucidate the epigenomic mechanisms of CS-induced lung disease.

We hypothesized that CS-induced genome-wide changes of histone modifications may contribute to CS-induced gene expression changes in human airway epithelium that are pathological to CS-related respiratory diseases. To characterize CS-induced chromatin changes at a genome-wide scale in human bronchial epithelium and infer plausible mechanisms, HBE cells were cultured at air-liquid interface (ALI) and exposed to the vapor phase of CS to model *in vivo* effects of CS in human bronchial epithelium. We then performed a systematic and unbiased global mapping of CS-induced H3K27Ac changes and assessed correlation of chromatin modification changes with sub-chronic CS-induced gene expression changes (day 1 to day 4) in human bronchial epithelial cells cultured at ALI.

## Results

### Establishment of air-liquid interface culture for normal human bronchial epithelial cells

**Figure 1:**
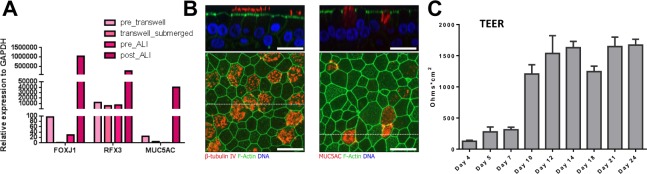
Establishment of air-liquid interface (ALI) to culture human bronchial epithelial (HBE) cells. (**A**) Expression of Forkhead Box J1 (FOXJ1), Mucin 5AC (MUC5AC) and Regulatory Factor X3 (RFX3) in HBE cells cultured at ALI at different stages measured by RT-PCR. Representative results from multiple repeats (N > 3) were shown here. (**B**) Pseudostratified architecture and differentiation of HBE cells cultured in ALI by immunofluorescnce staining for beta IV tubulin, MUC5AC, both in red and F-actin in green. Cell nuclei were stained by Hoechst. Side-view images (top panels) were reconstructed from z-stacks, and correspond to a z-slice along the dotted white line in the lower image. Top-view images (bottom panels) are maximum intensity projections over the height indicated by the bracket in the upper image. Scale bars are 20 µm. (**C**) Epithelial barrier integrity was quantified by TEER (trans-epithelial electrical resistance) measurements. Means ± SD are from 6 wells per time point.

To characterize CS-induced genome-wide epigenetic changes, we applied a previously described *ex vivo* model to culture normal human primary bronchial epithelial cells (HBE) from healthy donors at air-liquid interface (ALI). After 21 days of post-ALI culture, cells expressed significantly increased levels of FOXJ1 (foxhead box J1) (a marker for ciliated cells), RFX3 (regulatory factor X3) (a marker for ciliated cells) and MUC5AC (a marker for goblet cells), suggesting efficient differentiation of HBE cells at ALI (Fig. 1A). This *in vitro* model recapitulated the morphology of human bronchial epithelium as indicated by the organization of ciliated cells stained for beta IV -tubulin and goblet cells stained for MUC5AC into pseudo-stratified, well-differentiated epithelium^9^ (Fig. 1B). Cilia beating (Supplemental Video 1) and apical mucous secretion suggested functional similarity of this model with normal human airway epithelium. Increased levels of transepithelial electrical resistance (TEER) suggested increased maturity of the epithelial layer (Fig. 1C). Finally, the *in vitro* model of pseudostratified HBE cells allowed us to use mainstream cigarette smoke (CS) and expose cells to a complete vapor phase of CS^10^. The vapor phase of CS contains many active compounds that are typically lost in cigarette smoke extract, which is commonly used in undifferentiated cells maintained in submerged condition.

### Cigarette smoke exposure leads to a global genome reprogramming of H3K27Ac mark in HBE

In order to obtain genome-wide patterns of histone modifications in HBE in response to CS exposure, we applied chromatin immunoprecipitation followed by sequencing (ChIP-Seq) in HBEs cultured at ALI after exposure to CS. We chose 3 hours of CS exposure prior to ChIP-Seq to identify direct and driving events of chromatin remodeling that may contribute to subsequent transcriptomic changes. Specifically, H3K27Ac was used to interrogate active promoter and enhancer regions.

Using MACS (Model-based Analysis for ChIP-Seq)^11^ we detected 6589 peaks in the CS-exposed cells and 3462 peaks in the air-exposed cells at a significance cutoff of p < 1 × 10^−6^. Further analysis of these peaks using MAnorm^12^ allowed us to further classify them and identify 1881 “air-unique” peaks, 4997 “smoke-unique” peaks, and 1581 “common” peaks. The increased number of H3K27Ac peaks in the CS-exposed cells relative to the air-exposed cells likely indicates a rapid global chromatin relaxation triggered by CS exposure. This relaxation of condensed chromatin triggered by CS exposure corresponds to an increased number of accessible chromatin regions for transcription factor binding and subsequent gene activation.

### Impact of Chromatin Remodeling on Gene State

**Figure 2:**
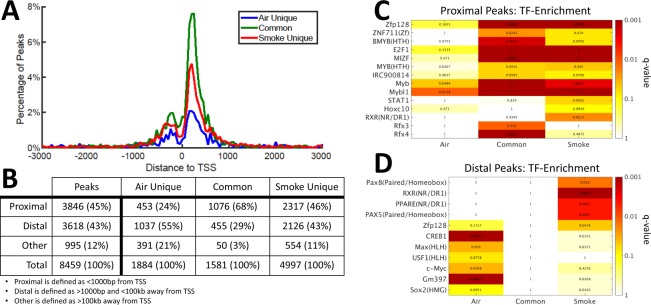
Analysis of H3K27Ac peaks in HBE exposed to air or cigarette smoke. (**A**) Distribution of the location of H3K27Ac ChIP-Seq peaks relative to the nearest transcription start site (TSS). (**B**) The number and percentage of each type of peak in a given group. (**C,D**) Transcription factor (TF) motif-enrichment analysis on peaks located in (**C**) proximal and (**D**) distal H3K27Ac peak regions. TFs were selected for display if they were enriched (fold-change > 1.1) and statistically significant (q-value < 0.1) in at least one peak-type. Numbers inside the figures are the q-values associated with a given pathway.

We next used HOMER (Hypergeometric Optimization of Motif EnRichment)^13^ to map each ChIP-Seq peak to a nearby gene. We then asked how far these H3K27Ac peaks are located from the transcription start site (TSS) of their associated genes (Fig. 2A). An overall enrichment of H3K27Ac peaks close to the TSS (within +/− 1 kb) was observed, with a dip directly over the TSS due to occupancy by RNA Polymerase II. Furthermore, a much higher percentage of common-peaks (68%) were located around the TSS than smoke-unique (46%) or air-unique peaks (24%). This may be in part caused by a higher robustness for the peaks that are identified in both air-exposed and smoke-exposed cells.

To further investigate CS-induced chromatin activation in human bronchial epithelium, we next subdivided the smoke-unique, air-unique and common peaks based on their proximity to the nearest gene (Fig. 2B); regions within 1 kb of a gene’s TSS were designated as “proximal” while those outside this region, but still within 100 kb of a gene’s TSS, were designated as “distal”. A small fraction (12%) of regions were extremely far away (>100 kb) from any annotated RefSeq TSS. We found that less than one-third (29%) of common-peaks were distally located, whereas smoke-induced (smoke unique peaks) and smoke-reduced (air unique peaks) peaks had 43% and 55% of peaks that were distally located, respectively (Fig. 2B). This suggests that CS-induced dynamic chromatin changes (opening or closing) tend to be farther from the gene transcription start site as compared to chromatin regions maintained stably between air and CS-exposure.

We also compared the locations of H3K27Ac activation peaks with regions enriched for published H3K4me3 and H3K27me3 in HBE cells exposed to cigarette smoke condensate;^8^ these histones mark active and repressed promoter regions, respectively. We found H3K27Ac peaks were almost exclusive to H3K27me3, a repressive mark for promoters. However, we do observe a high level of overlap between our 1581 “common” H3K27Ac peaks and published regions enriched for H3K4me3 in both air and smoke conditions after either 10 days or 10 months of exposure (Supplemental Fig. 1). This is consistent with our observation that “common” peaks are enriched near the TSS (see Fig. 2B).

To gain mechanistic insights into CS-induced chromatin remodeling, we further identified transcription factors that were statistically associated with these active chromatin regions by HOMER (see Methods). In proximal regions, several transcription factors were enriched in the common peaks, including MYB/B-MYB and E2F1, which are known to promote smoke-related cell proliferation in cancer cell lines^14,15^ (Fig. 2C). Interestingly, although statistically significant enrichment of TFs was found in proximal common peaks, this was not the case for distal common peaks (Fig. 2D). This suggests that most of the transcriptional rewiring of genes induced upon smoke exposure may result from distal regulation. Furthermore, air- and smoke-peaks were enriched with differential, non-overlapping transcription factors, suggesting that differential sets of transcription factors are responsible for activating and inactivating genes after CS exposure. For example, MAX and c-MYC were enriched in air-unique peaks, while PAX5, PAX8 and RXR were enriched in smoke-unique peaks (Fig. 2D). Among these transcription factors, the RXR TF family has been found to promote the lung repair process in a murine emphysema model^16^. This may indicate some compensatory and protective transcriptional programming is induced in the early stages of CS exposure in HBE.

### Functional Characterization of CS-induced ChIP-Seq peak-associated Genes

We next identified the top smoke- and air-unique peaks based on the MAnorm analysis (Supplemental Table 1). One of the top smoke-induced H3K27Ac peaks is located in the first intron of AKR1C3 gene (Aldo-Keto Reductase Family 1 Member C3) (Supplemental Fig. 2A). This gene was previously reported to be reduced by cigarette smoke condense^17^. Another endoplasmic reticulum located protein, encoded by the ERP44 gene, also showed induction^18^ after Tabaco treatment *in vitro*. Interestingly, H3K27Ac peaks nearby genes related with RNA splicing (SF1, splicing factor 1), cell tight junction (CLDN3, Claudin 3) and DNA repair (SLF2, SMC5-SMC6 Complex Localization Factor 2) showed reduction after smoke exposure (Supplemental Fig. 2B), consistent with smoke-induced genomic instability and loss of cellular tight junction in airway epithelium. We validated a set of smoke-induced H3K27Ac peaks by ChIP-PCR in primary HBE cells from another healthy donor, cultured in ALI followed by the same exposure (Supplemental Fig. 3).

**Figure 3:**
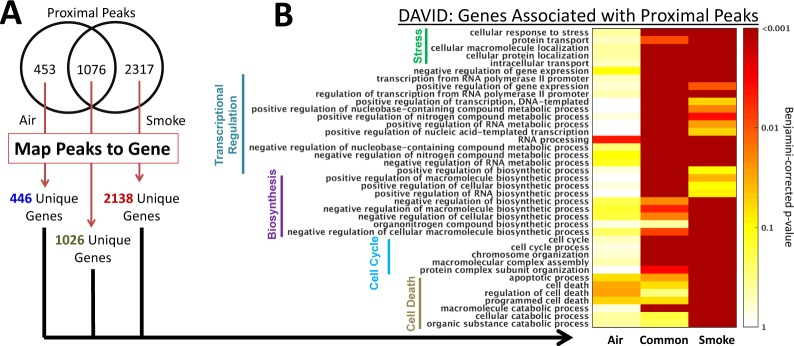
Functional enrichment of genes associated with H3K27Ac proximal peaks. (**A**) Venn diagram showing the number of proximal air-unique, smoke-unique or common peaks. These peaks were mapped to genes for pathway analysis. (**B**) A heat map showing the GO categories enriched in the three corresponding gene-sets by DAVID analysis. GO categories were selected for display if they were statistically-enriched at a Benjamini-Hochberg corrected p-value < 0.01 and at least 10% of the genes in the gene-set are also annotated to a certain GO category.

Next, we sought to collectively characterize the function of genes associated with H3K27Ac peaks upon CS exposure. We first analyzed proximal peaks and identified genes associated with each peak-type (“air-unique”, “smoke-unique” or “common”, see Fig. 3A). Since multiple H3K27Ac peaks may be associated with the same target gene, there is a small set of overlapping genes (N = 65) associated with both proximal air-unique peaks and smoke-unique peaks (Supplemental Fig. 4A). We applied DAVID pathway analysis to systematically assess the enrichment of Gene Ontology (GO) categories in each set of genes^19–21^ (Fig. 3B and Supplemental Table 2). Smoke-unique genes are enriched in biological pathways related with cellular stress response, macromolecular catabolic processes and cell death, consistent with activation of autophagy and apoptosis pathway by CS exposure^22–24^. We also observed marginal enrichment in pathways related to cell death for the air-unique and common genes (FDR = 0.03 and FDR = 0.06 for the “cell death” pathway, respectively). This may reflect noise in the dataset or cellular stress in the ALI model. However, importantly, this enrichment is far less significant than what we observe for the smoke-unique genes (FDR = 3.88 × 10^−7^).

We also performed pathway analysis on distally located peaks. In contrast to the proximally-identified gene sets, there is greater overlap of genes (N = 207) associated with distal air-unique and smoke-unique peaks (Supplemental Fig. 4B). In addition, despite a smaller number of significant pathways enriched with genes associated with distal-peaks compared to proximal peaks (Supplemental Fig. 4C, Supplemental Table 2), the majority of the significant functional-enrichment associations for distal-regions can be found in the smoke-unique peaks. Similar to the pathway analysis results in the proximal peaks, several of the GO-categories enriched in the smoke-unique distal peaks are related to “cell death” and “cell proliferation.” Overall, this analysis supports the notion that CS-exposure induces the *de novo* activation of pathways, rather than the removal of existing ones.

### Dynamic gene expression changes in human bronchial epithelium in response to cigarette smoke exposure

In order to assess the contribution of CS-induced chromatin modifications to subsequent CS-induced gene expression changes, we performed genome-wide expression profiling (microarray) and investigated whether the chromatin state of proximal and distal regulatory regions could predict the short-term and/or future transcriptional behavior of genes in response to CS exposure.

**Figure 4:**
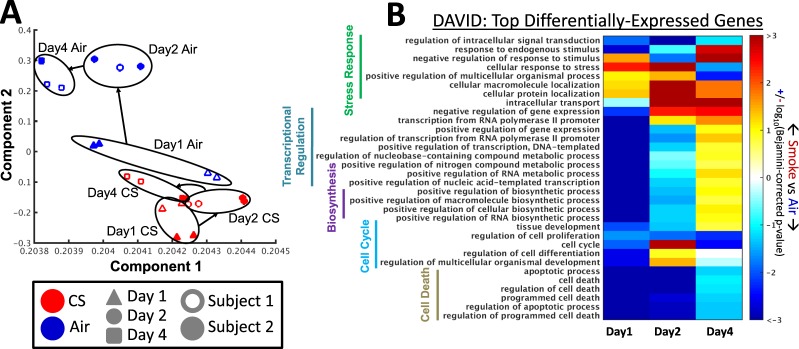
Gene expression analysis of HBE cultured at ALI and exposed to sub-chronic cigarette smoke. (**A**) Principal component analysis (PCA) showing the clustering of all samples in microarray analysis. Samples collected on the same day and condition are circled to help highlight their co-localization on the PCA plot. (**B**) A heat map showing the GO categories enriched in genes differentially expressed between smoke and air samples in each day. GO categories were selected for display if they were statistically-enriched at a Benjamini-Hochberg corrected p-value < 0.01 and at least 10% of the genes in the gene-set are also annotated to the GO category. Red colors indicate that genes associated with given GO category showed increased expression in smoke-exposed samples compared to air samples; blue colors indicate that genes associated with given GO category showed decreased expression in smoke-exposed samples compared to air samples.

Given that chronic CS exposure precedes onset of COPD in human patients, we choose a repeated CS-exposure model in HBE cells to mimic chronic CS exposure in human smokers. HBE cells (from two independent healthy donors) were cultured at ALI exposed to CS for different durations (Supplemental Fig. 5A). We then evaluated the expression of genes in air-exposed versus smoke-exposed cells at each of the three different time points (day 1, day 2 and day 4) (Supplemental Table 3). Principal component analysis showed clustering of air and smoke-treated samples, respectively (Fig. 4A). Furthermore, we also compared smoke-related gene expression changes in our *in vitro* HBE model with previously published gene expression changes in small airway epithelial cells (SAEC) from human smokers and non-smokers^25^. A significant portion differentially-expressed genes between air and smoke conditions at each of the three time points significantly overlap (p < 0.001 by Fisher’s Exact Test) with differentially-expressed genes in SAECs collected from smokers versus non-smokers (Supplemental Fig. 5B).

Additionally, by DAVID analysis, we identified Gene Ontology pathways that are significantly differentially-expressed between the smoke-exposed and the air-exposed cells on each day (Fig. 4B, Supplemental Table 4). On day-1 in smoke-exposed cells, we observed decreased expression of genes in GO-categories related to transcriptional regulation, but by day-2 and day-4 this trend had reversed with increased expression of these genes in smoke-exposed compared to air-exposed cells, suggesting gradual transcriptional regulation induced by smoke. However, stress response-related gene expression changes persisted from day 1 to day 4. These observations suggested differential trajectories of gene expression changes in response to CS exposure in human airway epithelium (Supplemental Figs 6 and 7). Using HBE cells from four healthy non-smoker subjects, we validated some of the observed gene expression changes induced by intermittent smoke exposure using RT-PCR in HBE cells maintained in ALI culture (Supplemental Fig. 8A,B).

Based on these observations, we applied linear mixed models to identify genes that showed smoke treatment interaction with exposure duration. Interestingly, 30 genes showed significant interaction between treatment and time (FDR < 0.1, Supplemental Table 5), suggesting that expression of these genes in response to smoke exposure depends on exposure duration in bronchial epithelial cells. We compared these 30 genes to publicly available HBE expression data collected via bronchial brushings in former and current smokers with and without COPD^26^. We found that 7 of these 30 genes are also differentially-expressed with respect to COPD status and 14 genes are differentially-expressed between former and current smokers; after controlling for these two variables (Supplemental Table 5);, none of these genes were significantly differentially-expressed with respect to pack-years.

### Integrated analysis on cigarettes smoke-induced dynamics of regulatory elements in human airway epithelium

**Figure 5:**
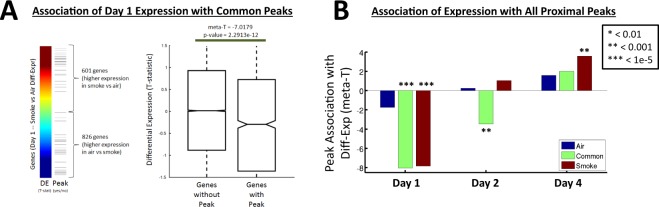
Differential-expression of genes associated with H3K27Ac peaks. (**A**) Illustrative example quantifying the association of differentially expressed genes upon CS exposure with proximal H3K27Ac peaks identified as common to smoke- and air-exposed cells. The left panel shows the value of the t-statistics across all genes (color bar) and the genes that are annotated with a proximal common peak (ticks). The right panel shows box plots (median and inter-quartile range) of the t-statistic values for genes that have a proximal common peak (indicated by ticks in the left panel) and all other genes. The significance in the difference of these distributions is indicated. (**B**) The statistical association of the genes containing proximal H3K27Ac peaks with the differential-expression levels of genes in smoke compared to air exposed samples.

To understand how CS-induced dynamic changes of chromatin state may be mediating gene expression alterations upon CS-exposure, we next evaluated if the subsequent expression levels of the genes were associated with H3K27Ac localization. As illustrated in Fig. 5A, we sorted differentially expressed genes based on their t-statistic values indicating differential expression between CS-exposed and air-exposed samples on day-1. We then determined which genes were located proximal to H3K27Ac common-peaks (see also Fig. 2A), and compared the distribution of the t-statistic values for these genes with the distribution of the t-statistic values for all other genes. We found a significant overall decrease in the expression levels of genes nearby common-peaks upon smoke exposure, with an associated meta-T statistic value of −7.018 (p = 2.29 × 10^−12^).

We repeated this analysis for genes differentially expressed on each day (day-1, day-2, day-4, Fig. 4B) and genes associated with each peak-type (air-unique, smoke-unique, common, see Fig. 3A). Figure 5B shows the associated meta-T statistic values for each of these comparisons. Interestingly we observe on day-1 that the genes associated with H3K27Ac chromatin marks have an overall lower expression level in CS-exposed compared to air-exposed cells, yet over time we observe an increasing expression trend, and by day-4 the same set of genes showed increased expression in CS- versus air-exposed cells (Fig. 5B). This is true for genes associated with each of the peak-types, however, it is most significant for the genes associated with smoke-unique H3K27Ac peaks, suggesting that earlier open chromatin may predict sub-chronic gene expression changes later. This same trend was also observed when analyzing the expression data from each of the two subjects separately (Supplemental Fig. 9), indicating that the observed changes are likely independent of genotype. Two of the genes near smoke-induced ChIP-Seq peaks, including AKR1C3 (Supplemental Fig. 2; Supplemental Table 1) and TXNRD1 (Thioredoxim Reductase 1), also demonstrated significantly increased gene expression over multiple days as revealed by RT-PCR in four independent subjects-derived HBE cells cultured at ALI (Supplemental Fig. 8C).

## Discussion

Cigarette smoke (CS) is the most common risk factor for COPD and lung cancer. It has been shown that upon exposure to CS, bronchial epithelial cells demonstrate a series of cellular phenotypic changes accompanied by gene expression changes^27^. Additionally, recent progress in epigenetic profiling has revealed the molecular switches that regulate gene transcription. In light of these new findings, we first attempted to characterize epigenetic changes of human airway epithelium in response to cigarette smoke by genome-wide ChIP-Seq profiling of H3K27Ac. We also generated time series transcriptomic data in CS-exposed HBE cultured at air-liquid interface (ALI), an *in vitro* 3-D model that recapitulates sub-chronic CS exposure in human airway epithelium. Comparative analysis of H3K27Ac regions with time-series gene expression changes in HBE suggested CS-induced opening of chromatin leads to subsequent CS-induced gene transcriptional activation. Our data provides epigenome-wide insights into the chromatin states induced by CS exposure in the human bronchial epithelium.

Normal human bronchial epithelium, which is composed of ciliated cells, mucous producing goblet cells and basal cells, forms a natural line of defense against cigarette smoke-induced injury through a series of adaptations including global gene expression changes upon chronic CS exposure. However, COPD and lung cancer usually develop after decades of CS exposure. Recapitulating such chronic CS exposure *in vitro* is very challenging for a number of reasons: (1) it is impractical to expose cells to CS *in vitro* for an extended period comparable to CS exposure in patients for decades; (2) characteristic changes in a single cell type cultured in a mono-layer may be non-representative of *in vivo* changes due to lack of cell-cell communication; (3) without time-lapsed gene expression profiling, separation of primary effects from secondary effects of CS at best relies on speculation; (4) cigarette smoke extract generated by bubbling vapor phase of CS into culture medium lacks the complexity of whole cigarette smoke. To overcome these obstacles, we applied cigarette smoke exposure to HBE cultured at ALI where human bronchial epithelial cells were fully differentiated into pseudo-stratified airway epithelium composed of multiple epithelial cell types. We also applied intermittent CS exposure for 4 days to human bronchial epithelial (HBE) cells cultured via ALI to recapitulate exposure of epithelium to CS in human smokers. This exposure duration is long enough to reveal adaptive changes after sub-chronic CS exposure but still short enough before appearance of overall cellular phenotypic changes that may involve secondary gene expression changes. On every day of CS exposure, we consistently observed increased expression of genes that are known to respond to oxidative stress, such as HMOX-1^28^ and OKL38^29^ (Supplemental Figs 6 and 8), suggesting a rapid and protective adaptation in HBE responding to CS. In contrast, cell cycle genes such as CCNB2 and ER-stress genes such as SYVN1 (Synovial Apoptosis Inhibitor 1, Synoviolin)^30,31^ showed delayed induction by CS on day 4, suggesting pathways related to protein degradation respond only after prolonged CS exposure (Supplemental Fig. 7). More importantly, many of the 30 genes that showed interactions between smoke exposure and duration (Supplemental Table 5) are associated with membrane lipid trafficking in response to CS exposure. This may indicate that prolonged CS exposure to the lung epithelium causes significant injury and damage which is handled by remodeling and “resealing” damaged areas of the cell membrane^32^. Furthermore, nearly half of the 30 smoke treatment and duration interaction genes that we identified in this ALI *in vitro* model were associated with current smoking status in human smokers (Supplemental Table 5).

Cigarette smoke, composed of approximately 100 compounds that present health risks^33^, can cause both irreversible DNA damage (mutations) and reversible changes to the epigenetic landscape of bronchial epithelium^7^, including histone modification of the bronchial epithelium (BE). Epigenetic modification of the HBE in response to CS exposure may confer adverse cellular consequences that contribute to the pathogenesis of common lung diseases, including COPD, asthma and lung cancer. However, it remains unclear how CS exposure changes the epigenomic landscape on a genome-wide scale. ChIP-Seq has enabled development of detailed histone maps in diverse cell types. The ENCODE consortium generated ChIP-Seq data for dozens of histone marks and TFs across a wide range of cell lines, and several primary cell types^34–37^, providing a starting point for characterization of the epigenome in health and disease. Our study aimed to directly address the epigenome-wide changes of a specific chromatin modification in response to CS exposure in human airway epithelium, and to provide mechanistic insights into CS-induced gene expression changes related to this chromatin modification. Through motif analysis on H3K27Ac peak regions, we identified a series of TFs that may point to novel regulatory mechanisms. For example, we found that H3K27Ac peaks are enriched with B-myb binding sites, which is consistent with increased expression of B-myb induced by cigarette smoke in immortalized endocervical cells^38^. Expression of E2F, another TF enriched in the smoke unique H3K27Ac peaks, is induced by cigarette smoke extract (CSE) in pulmonary artery smooth muscle cells, which may be related to the pulmonary arterial hypertension complications observed in COPD patients^39^. Most importantly, CSE treatment has been shown to increase binding of Egr-1 and E2F to regulate the autophagy gene LC3B through decreasing HDAC activity, which in turn activates the autophagy pathway and promotes smoke-induced emphysema^40^.

We also recognize a few limitations of our current model: (1) The lung is a complex organ composed of more than 40 different cell types. Using well-differentiated bronchial epithelial cells cultured in ALI condition provides physiological advantages over undifferentiated airway epithelial cells cultured in submerged condition. However, it lacks other lung cell types such as immune cells and fibroblasts as well as minimal interaction with matrix, which may potentially have important effects on the chromatin state of epithelial cells induced by cigarette smoke exposure *in vivo*. (2) As clearly demonstrated in a recent publication, epithelial cells from the trachea, large airway, and small airway all have distinct gene expression patterns^25^. Although our model can capture smoke-induced changes that are shared between the large and small airway, chromatin and gene expression changes that are unique to small airway epithelial cells may be missed. This may partially explain why only ~10% differentially-expressed genes in our model overlapped with previously published SAEC smoke-related genes (Supplemental Fig. 5B). (3) We also recognize that the treatment duration of our *in vitro* model, 4 days of intermittent smoke exposure, is distinct from decades of *in vivo* smoke exposure in human COPD subjects. Almost half of our of the 30 interaction genes were also differentially-expressed based on smoking status in a public human data set^26^ (Supplemental Table 5). (4) Well-differentiated airway epithelial cells contain multiple cell types: ciliated cells, goblet cells, and basal cells. Single cell RNA-Seq and open chromatin mapping methods^41,42^ developed recently will enable assignment of smoke-induced chromatin modification and gene expression changes to specific airway epithelial cell type.

Herein, we reported genome-wide chromatin modifications induced by CS exposure in normal human airway epithelium cultured at air-liquid interface. Additionally, we linked such epigenetic modifications in human airway epithelium with dynamic gene expression changes in response to sub-chronic CS exposure. By characterizing CS-induced H3K27Ac changes at a genome-wide scale, we demonstrated the downstream consequences of these changes on transcriptional activity in human airway epithelium. Further mechanistic characterization of these histone modifications will provide a better understanding on the pathogenesis of CS-induced lung disease.

## Methods

### Culture of primary human bronchial epithelial cells

Normal Human Bronchial Epithelial cells (NHBE) from healthy non-smoking donors (N = 2) purchased from Lifeline Cell Technology (Frederick, MD) were used for Chip-Seq and microarray analysis. HBE cells from additional two healthy non-smoking donors were used for RT-PCR and ChIP-PCR validation. Details on each subject are included in Supplemental Table 6. The passage number of NHBE cells used for air-liquid interface test was less than one passage after receiving cells from the vendor (typically below passage 3). Cultures were incubated at 37 °C in a humidified 5% CO_2_/95% air atmosphere and the medium was changed every other day.

### Air-liquid interface culture of HBE cells

For an ALI culture, HBE cells were seeded on Transwell^®^ inserts (#3460, Corning Incorporated, Corning, NY). HBE cells were seeded at a density of 1 × 10^5^ cells/1.1 cm^2^ and maintained in submerged with BEGM medium. After the cells reached 100% confluence, the cultures were airlifted by removing BEGM medium and feed from basal chamber only with PneumaCult^TM^-ALI medium which is composed of PneumaCult^TM^-ALI basal medium (05002, StemCell Technology, Vancouver, BC) supplemented with PneumaCult™-ALI 10X Supplement (05003, StemCell Technology, Vancouver, BC) and PneumaCult™-ALI Maintenance Supplement (100X) (05006). The cells are well-differentiation after 28 days post airlift.

### Immunofluorescence Staining in HBE cells cultured in ALI

On day 21 of air-liquid interface culture, HBE cells in the transwell were fixed in 4% paraformaldehyde for 1 hour at room temperature and then were permeabilized with 0.02% Triton-X in PBS (PBST) for 15 minutes and incubated in a blocking solution (10% normal goat serum and 1% BSA in PBST) for 1 hour. Cells were then incubated with primary antibody (anti beta-IV tubulin, Sigma, 1:500 or anti MUC5AC (clone 45M1), Thermo Scientific, 1:500) overnight at 4°C. After incubation of secondary antibody for 1 hour at room temperature, cells were counterstained with Hoescht (1:5000) for 10 minutes at room temperature before imaging. Cell images were obtained on a Zeiss Axio Observer Z1 equipped with an Apotome module and processed using Zen software.

### Cigarette smoke exposure at ALI culture

For cigarette smoke exposure, a smoking chamber maintained at 37 °C was used. Primary human bronchial/epithelial cells (HBE) cultured at ALI were exposed to mainstream smoke from one 3R4F research cigarette (University of Kentucky, Lexington, KY) for 10 minutes and then incubated in a humidified 5% CO_2_/95% air atmosphere with smoke for 3 hours to allow for progression of the smoke reaction. After cigarette smoke exposure, the cell plates were moved to the normal conditioned cell culture incubator without smoke for further culture. Intermittent CS exposure was performed at exactly the same time on every day until collection time.

### Chromatin immunoprecipitation (ChIP) followed by next generation sequencing

After 21 days of ALI culture and confirmed fully differentiation, HBE cells were exposed to either Air or CS for 3 hours. Duplicate wells were used for each condition. Cells harvested from ALI culture were cross-linked by 1% formaldehyde (F79–500, Fisher, Waltham, MA) for 10 minutes followed by neutralization with 2.5 M glycine. After washing with PBS twice, cells were then lysed by sonication for 5 cycles (“5 seconds on 10 seconds off” module). Cell lysates were then incubated with 5 μg H3K27Ac antibodies at 4 °C overnight. On the second day, protein A/G Dynal beads were washed three times in cold PBS supplemented with BSA (5 mg/ml). Immunoprecipitated chromatin was then collected after incubation with beads for 6 hours at 4 °C. Dynal beads were washed with low salt buffer twice, high salt buffer, LiCl buffer and TE buffer twice sequentially. Elution buffer was then added to each sample and beads were heated in 65 °C overnight for reverse-crosslinking. On the third day, samples were treated with RNaseA and protease K sequentially, DNA products were purified by MiniElute PCR Purification Kit from QIAGEN. DNA was measured by Picogreen for double strand DNA. To generate sequencing library, samples were prepared using Next ChIP-Seq Sample preparation kit (E6200S, NEB, Ipswich, MA). Libraries were sent to Harvard Medical School sequencing facility to apply in Illumina Hi-Seq. 2500 sequencer at 50 bp pair-end for sequencing.

### ChIP-Seq data analysis

After initial QC of sequencing data, we then used the MACS (Model-based Analysis for ChIP-Seq) program to identify peaks enriched for the H3K27Ac chromatin mark at a cutoff of p < 1 × 10^−6^. In order to quantitatively identify CS or Air specific ChIP-Seq peaks, we applied MAnorm^12^ to identify “air-unique” peaks, “smoke-unique” peaks and “common” peaks.

### Comparison of H3K27Ac Peaks with H3K4me3 and H3K27me3

Raw bed files with read counts for H3K4me3 and H3K27me3 histone marks measured in bronchial epithelial cells exposed to air and smoke conditions for different durations (10 days and 10 months)^8^ were downloaded from the gene expression omnibus (GSE103331). Regions with more than a 10 reads in each sample were identified. The bedtools^43^ intersect function was then used to compare overlap our “air-unique”, “smoke-unique” and “common” H3K27Ac peaks with these regions. For each histone mark (H3K4me3 and H3K27me3) we determined the percentage of each of our peak-types that overlapped with the mark in the air-exposed sample, smoke-exposed sample, both the air-exposed and smoke-exposed samples, or neither. These percentages are shown as a stacked bar graph in Supplemental Fig. 1.

### ChIP-Seq peak-analysis and motif discovery analyses

We used HOMER (Hypergeometric Optimization of Motif EnRichment)^13^ to annotate and analyze each of the three sets of ChIP-Seq peaks (smoke-unique, air-unique and common). More specifically, we used HOMER’s “annotatePeaks.pl” function to map each peak to the nearest gene based on the location of RefSeq annotated transcriptional-start-sites (TSS). We then further divided each of the peak-types based on their proximity to annotated TSS. This created nine total sets of peaks: “proximal”, “distal” and “remote” for either the “smoke-unique”, “air-unique” or “common” condition. We applied HOMER’s “findMotifsGenome” function to investigate the enrichment for known motifs in each of these nine sets of peaks separately. For visualization, we selected TFs that were found in at least 5% of peaks, were enriched at a FC (fold change) >1.1 and had an FDR significance <0.1, in at least one of the peak-types being investigated (e.g. for Fig. 2C, we included TFs that met this criteria in either the air-unique, common, or smoke-unique proximal peaks).

### Pathway analysis on genes associated with H3K27Ac peaks

Pathway analysis for genes associated with ChIP-Seq peaks was performed using the DAVID (Database for Annotation, Visualization and Integrated Discovery; https://david-d.ncifcrf.gov/). Genes associated with a given peak-type (smoke-unique, air-unique, common) and relative distance to a TSS (proximal or distal) were selected and submitted to the DAVID online tool and all Gene Ontology (GO) biological process pathways were analyzed, resulting in six total DAVID analyses run on the ChIP-Seq data. Data were then downloaded and results merged. To visualize the results in Fig. 3B, we selected only more specific GO-categories (those with fewer than 2000 total members) that were enriched in at least one of the gene sets at a Benjamini-Hochberg corrected p-value less than 0.01 and for which at least 10% of the peak-associated genes were represented in the category.

### Microarray data analysis

For gene expression profiling experiments in HBE cells cultured at ALI, cells from two independent subjects with two technical repeats (two independent transwells for each condition) were used. CS treatment for different duration was performed as indicated in Supplemental Fig. 5A. RNA samples were extracted using RNAeasy Kit from each condition and gene expression profiling was performed using one Illumina HumanHT-12_V4_0_R2_15002873_B chip. Regular data QC check was performed as previously described^44^. Gene expression levels for the two subjects were then batch-corrected using ComBat in the sva Bioconductor package^45^. Differential expression levels between CS-exposed vs air-exposed samples were evaluated separately for day-1, day-2 and day-4 samples using a student *t*-test.

To detect genes that showed interactions between smoke treatment and treatment time, for each gene probe, we ran the linear mixed effects model ${y}_{ij}={\beta }_{0i}+{\beta }_{1}tr{t}_{i}+{\beta }_{2}tim{e}_{j}+{\beta }_{3}tr{t}_{i}$ * $tim{e}_{j}+{e}_{ij},i=1,\ldots ,$ $n,j=1,2,3,$ where n is the number of subjects, regression coefficient ${\beta }_{0i}$ is the random effect following normal distribution, $tim{e}_{1}=1$ day cs exposure, $tim{e}_{2}=2$ day cs exposure, $tim{e}_{3}=4$ day cs exposure, and regression coefficients ${\beta }_{1}$, ${\beta }_{2}$, and ${\beta }_{3}$ are fixed effects. We are interested in testing if ${\beta }_{3}=0$ or not. An interaction effect is declared as significant if FDR adjusted p-value for testing ${\beta }_{3} < 0$.

### Comparison with Bronchial Brushing Expression Data

Raw gene expression data from^26^ was downloaded from the gene expression omnibus (GSE37147) and rma-normalized using the *affy* library^46^ in R together with a custom CDF mapping probes to Entrez gene-ids^47^. Differential expression was evaluated using the *limma* package^48^ with a linear model y ~1 + PY + Smk + copd, where “PY” is the pack-years of smoking, “Smk” indicates whether a subject is a current or former smoker, and “copd” indicates whether the subject has COPD. In total, 222 subjects from the public data set that had values for all three variables in the phenotype file were analyzed.

### Pathway analysis on differentially expressed genes

Pathway analysis for genes associated with differentially-expressed genes was performed using the DAVID (Database for Annotation, Visualization and Integrated Discovery; https://david-d.ncifcrf.gov/). Genes differentially-expressed at p < 0.05 for each comparison (CS-exposed vs air-exposed for day-1, day-2 and day-4) were selected and submitted to the DAVID online tool and all GO biological process pathways were analyzed. Genes with increased expression were evaluated separately from those with decreased expression, resulting in six total DAVID analyses run on the expression data. To visualize the results, we selected only more specific GO-categories (those with fewer than 2000 total members) that were enriched in at least one of the gene sets at a Benjamini-Hochberg corrected p-value less than 0.01 and for which at least 10% of the differentially-expressed genes in the set were represented in the category.

### Comparison with Small Airway Epithelium Expression Data

Raw gene expression data from^25^ was downloaded from the gene expression omnibus (GSE64614) and rma-normalized using the *affy* library^46^ in R together with a custom CDF mapping probes to Entrez gene-ids^47^. 108 small airway epithelium samples from smokers with COPD were compared with 60 small airway epithelium samples from healthy smokers using a t-test. This identified 2513 genes that were differentially-expressed at p < 0.001 that were also measured in our array experiments. This p-value cutoff was selected so that the number of differentially-expressed genes identified from this public data was similar to the number of genes identified as differentially-expressed on each day in our analysis.

### ChIP-PCR and RT-PCR validation

In order to validation our initial ChIP-Seq and microarray analysis, we performed qPCR validation on selected H3K27Ac ChIP-Seq peaks or differentially expressed genes after intermittent smoke exposure as described previously^44,49^.

### Integration of gene expression and ChIP-Seq peaks

To understand how chromatin states may be mediating expression alterations upon CS-exposure, we evaluated the expression levels of the genes associated with each of our peak-regions. We performed a t-test that compared the t-statistic values for genes associated with a given peak-type, to the t-statistic values for all other genes. The t-statistic values were taken from the differential expression analysis as described above. This included differential expression analysis of CS-exposed vs air-exposed samples separately for day 1, 2, and 4. H3K27Ac associations were evaluated for proximal smoke-unique, proximal air-unique and proximal common peaks. Figure 5B shows the associated meta-T statistic values for each of these nine comparisons.

## Electronic supplementary material

Supplemental Video

Supplemental Information

Supplemental Table 2

Supplemental Table 3

Supplemental Table 4
