# Supplementary material for: Integrative epigenomic analysis in differentiated human primary bronchial epithelial cells exposed to cigarette smoke
Source: Sci Rep. 2018 Aug 24;8:12750. doi: 10.1038/s41598-018-30781-3 (PMC6109173; doi:10.1038/s41598-018-30781-3)
Supplement: Supplementary file 2 — Supplemental Information [file 41598_2018_30781_MOESM2_ESM.pdf]

Supplemental Material For:

Integrative epigenomic analysis in differentiated human primary bronchial epithelial cells exposed to cigarette smoke

Kimberly Glass[1], Derek Thibault[1], Feng Guo[1], Jennifer A. Mitchel[2], Betty Pham[1], Weiliang Qiu[1], Yan Li[1], Zhiqiang Jiang[1], Peter Castaldi[1,3], Edwin K. Silverman[1,3], Benjamin Raby[1,3], Jin-Ah Park[2], Guo-Cheng Yuan[4,5] and Xiaobo Zhou*[1,3]

[1]Channing Division of Network Medicine, Brigham and Women's Hospital and Harvard Medical School
[2]Department of Enviromental Health, Harvard T.H. School of Public Health
[3]Division of Pulmonary and Critical Care Medicine, Department of Medicine, Brigham and Women's Hospital and Harvard Medical School.
[4]Department of Biostatistics and Computational Biology, Dana-Farber Cancer Institute
[5]Department of Biostatistics, Harvard T.H. School of Public Health

*Correspondence: xiaobo.zhou@channing.harvard.edu

Table of Contents:

Supplemental Figure 1 2
Supplemental Figure 2 2
Supplemental Figure 3 3
Supplemental Figure 4 3
Supplemental Figure 5 4
Supplemental Figure 6 4
Supplemental Figure 7 5
Supplemental Figure 8 5
Supplemental Figure 9 6
Supplemental Table 1 7
Supplemental Table 2 9
Supplemental Table 3 9
Supplemental Table 4 9
Supplemental Table 5 10
Supplemental Table 6 11
Supplemental Video 11
Supplemental References.......... 11

Supplemental Figures

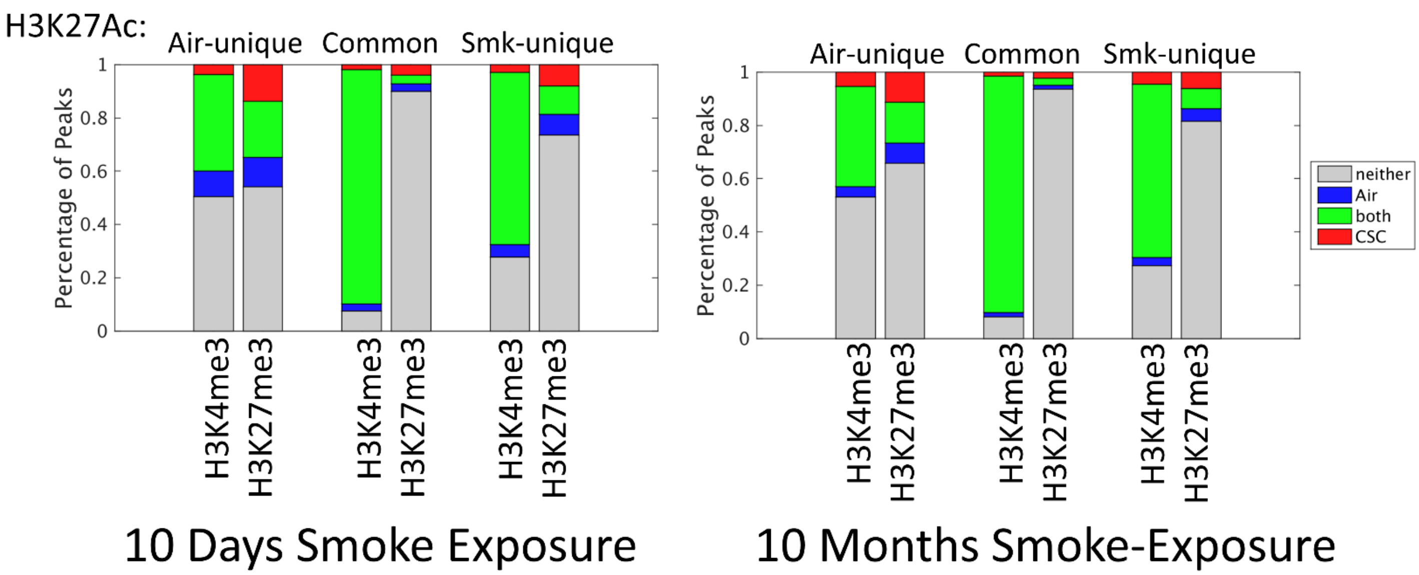

Supplemental Figure 1. The percentage of each of the three H3K27Ac peak-types (air-unique, common, smoke-unique) that overlap with regions marked by H3K4me3 or H3K27me3 in samples exposed to air and smoke conditions for two durations: 10 days (left panel), or 10 months (right panel) from a published paper [1].

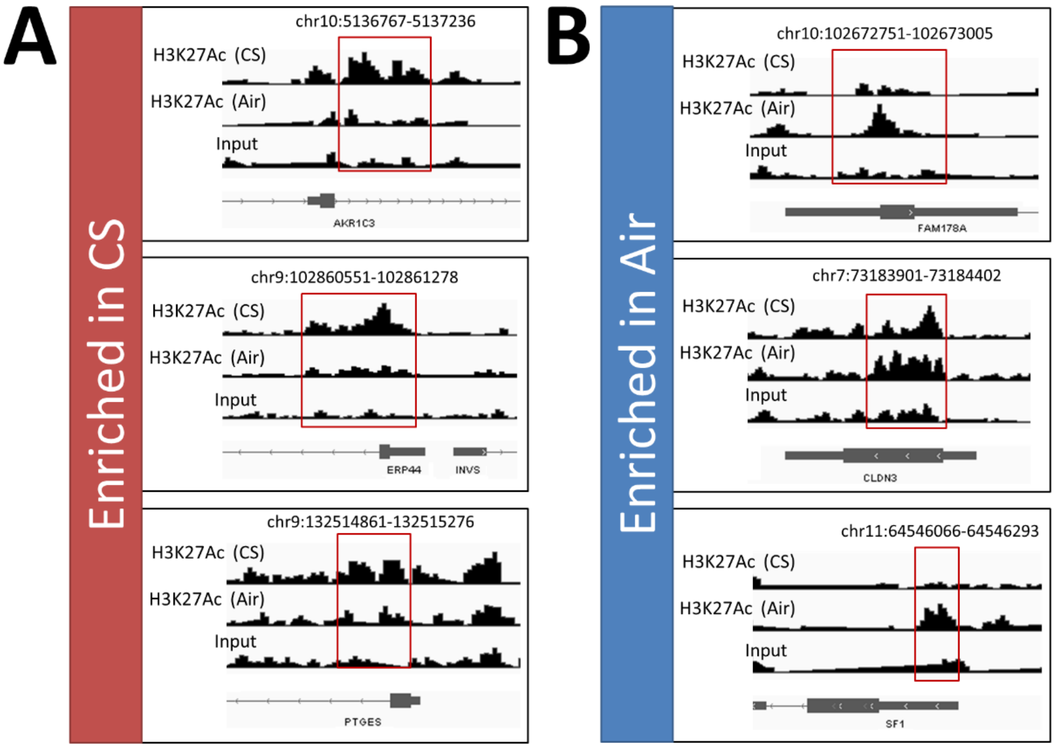

Supplemental Figure 2: Several representative **(A)** smoke-unique and **(B)** air-unique H3K27Ac peaks and their nearby genomic annotations.

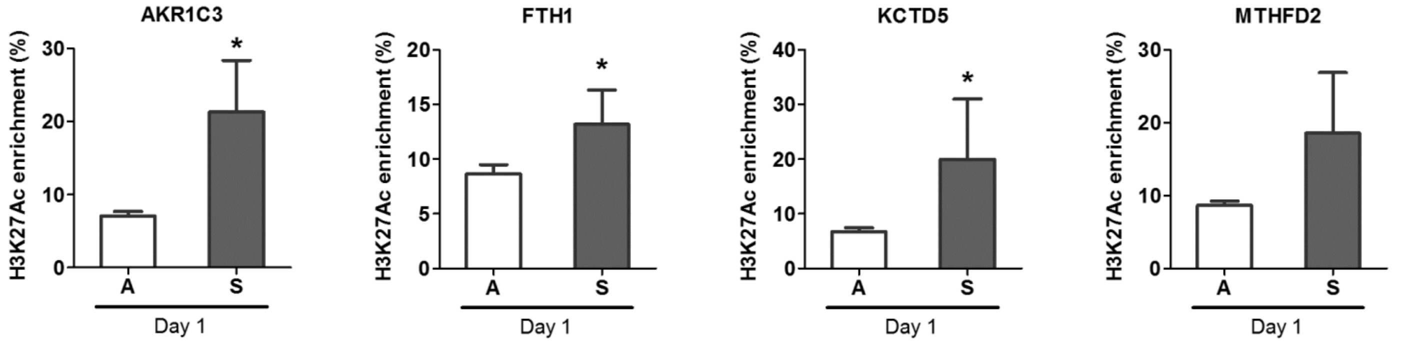

Supplemental Figure 3. Validation of ChIP-Seq by ChIP-PCR in HBE cells from another independent healthy donor. Relative enrichment of H3K27Ac in HBE cells cultured in ALI with 3 hours of either air or smoke treatment detected by ChIP-PCR. Names of closest genes are indicated on the top of the graph. A: Air, S: Cigarette Smoke. Mean+/-SD are from two technical repeats. * p<0.05.

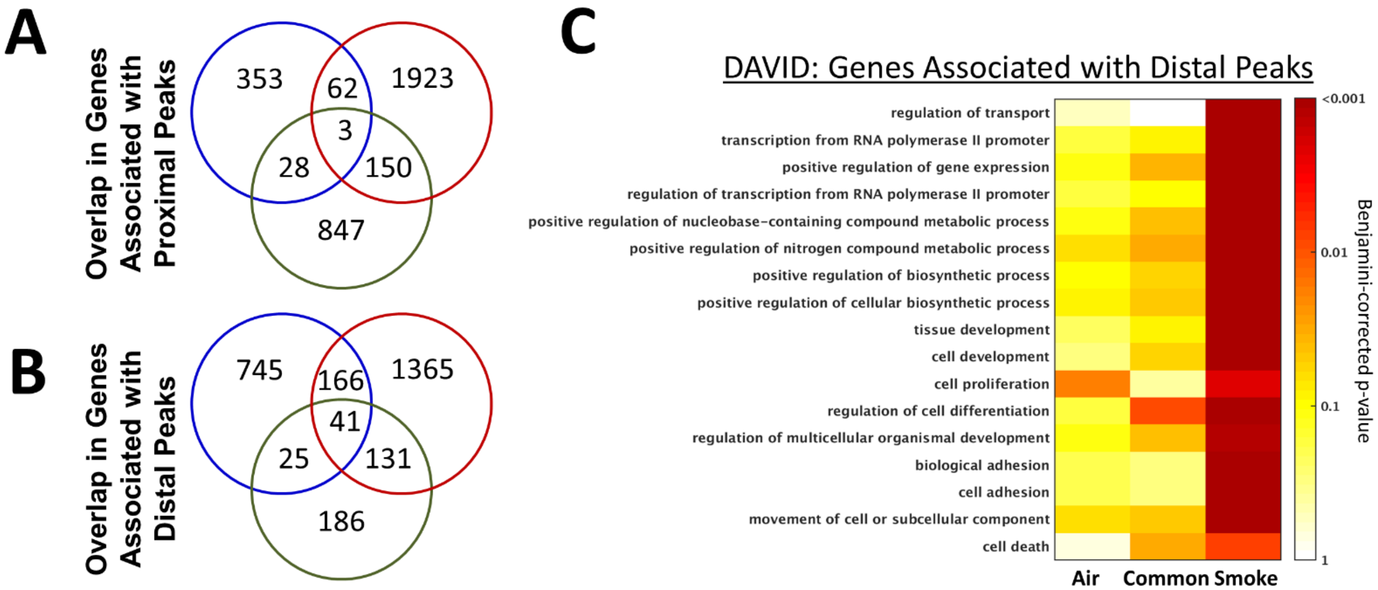

Supplemental Figure 4. Functional and expression analysis using sets of genes that are associated with peaks located distally from the nearest transcription start site (TSS). Venn diagrams illustrating the overlap in genes associated with the air- (blue circle), smoke- (red circle) and common- (green circle) unique H3K27Ac peaks, either those that are **(A)** located proximal to the promoters, or **(B)** distally located from the promoters. **(C)** A heat map showing the GO categories enriched in gene-sets derived from distally-located air-unique, common, and smoke-unique peaks. GO categories were selected for display if they were statistically-enriched at a Benjamini-Hochberg corrected p-value < 0.01 and at least 10% of the genes in the gene-set are also annotated to the GO category.

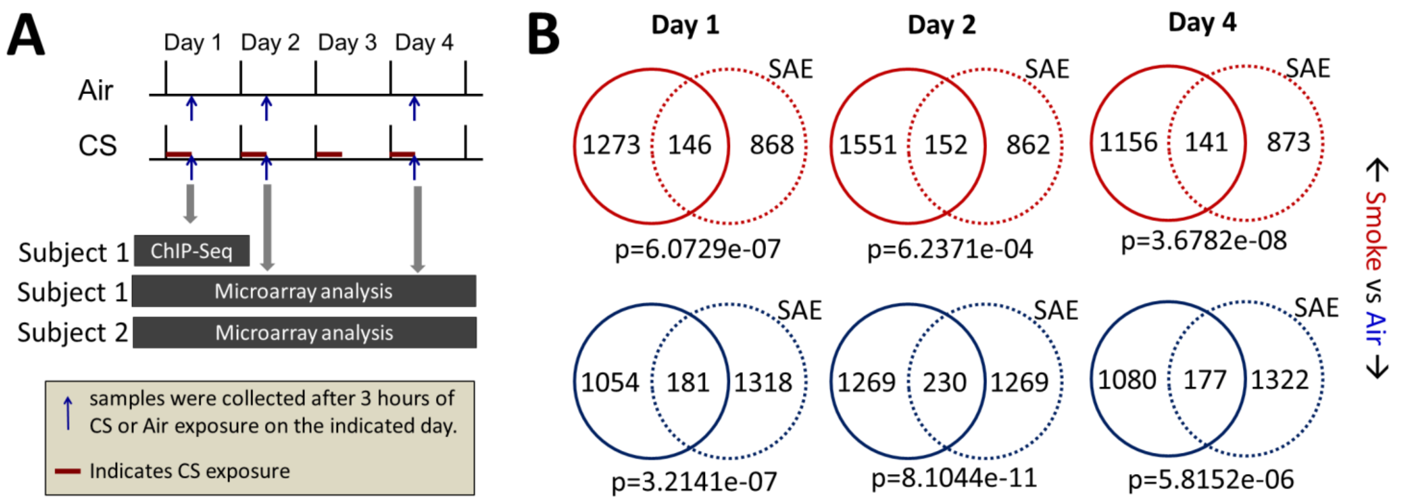

Supplemental Figure 5: (A) Experimental design for the sample collection and smoke treatment for gene expression profiling in human bronchial epithelial cells cultured at air-liquid interfaces. One subject is shared between ChIP-Seq and microarray experiments. **(B)** Overlap between the genes differentially-expressed with either increased (red) or decreased (blue) expression in smoke compared to air samples on each of three days (day 1, 2, and 4; solid circles), and genes that are differentially-expressed in small airway epithelium collected from smokers and non-smokers (dashed circles). Note that the set of genes in the dashed circles is the same across the comparisons.

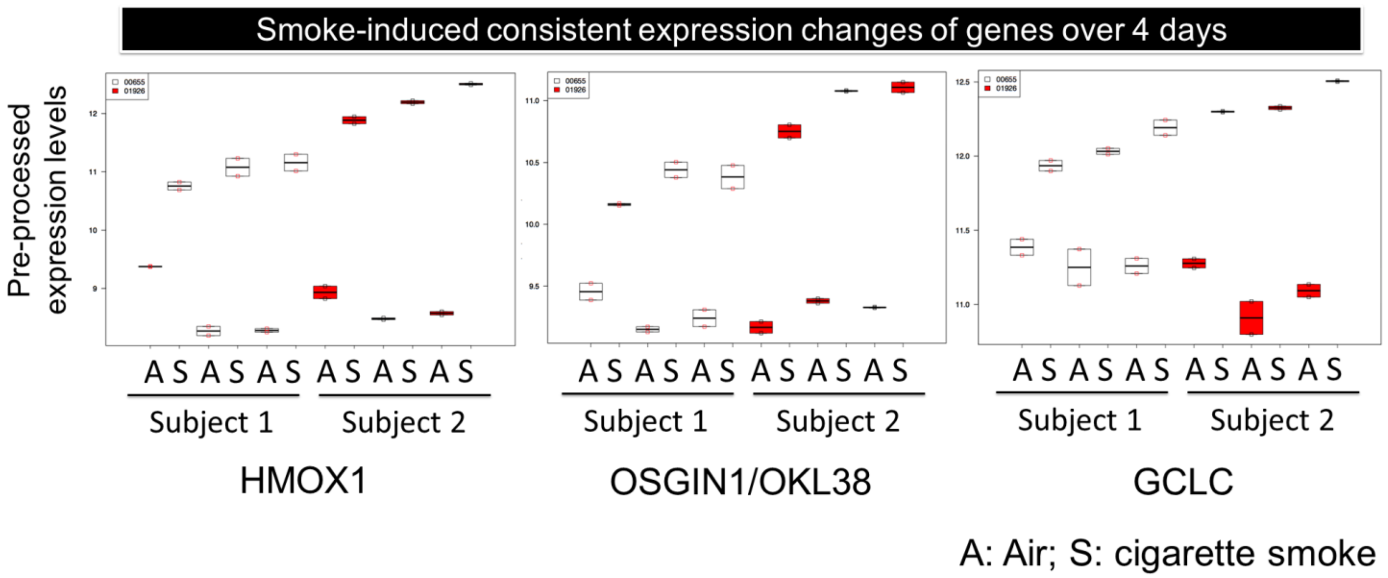

Supplemental Figure 6. Representative examples of genes that showed consistent expression changes over 4 days of cigarette smoke exposure in HBE cells derived from two subjects cultured at ALI. Box plot is generated using pre-processed expression levels of each gene from duplicate wells in each condition**.**

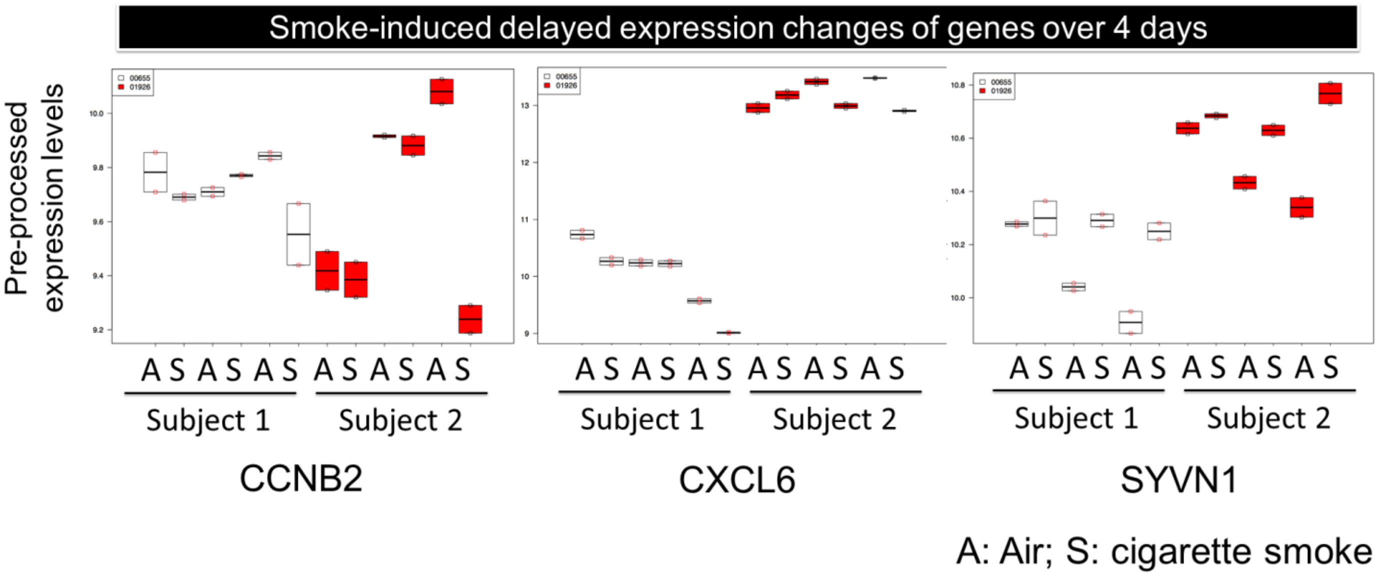

Supplemental Figure 7. Representative examples of genes that showed delayed expression changes in samples after 4 days of cigarette smoke exposure in HBE cells derived from two subjects cultured at ALI. Box plot is generated using pre-processed expression levels of each gene from duplicate wells in each condition**.**

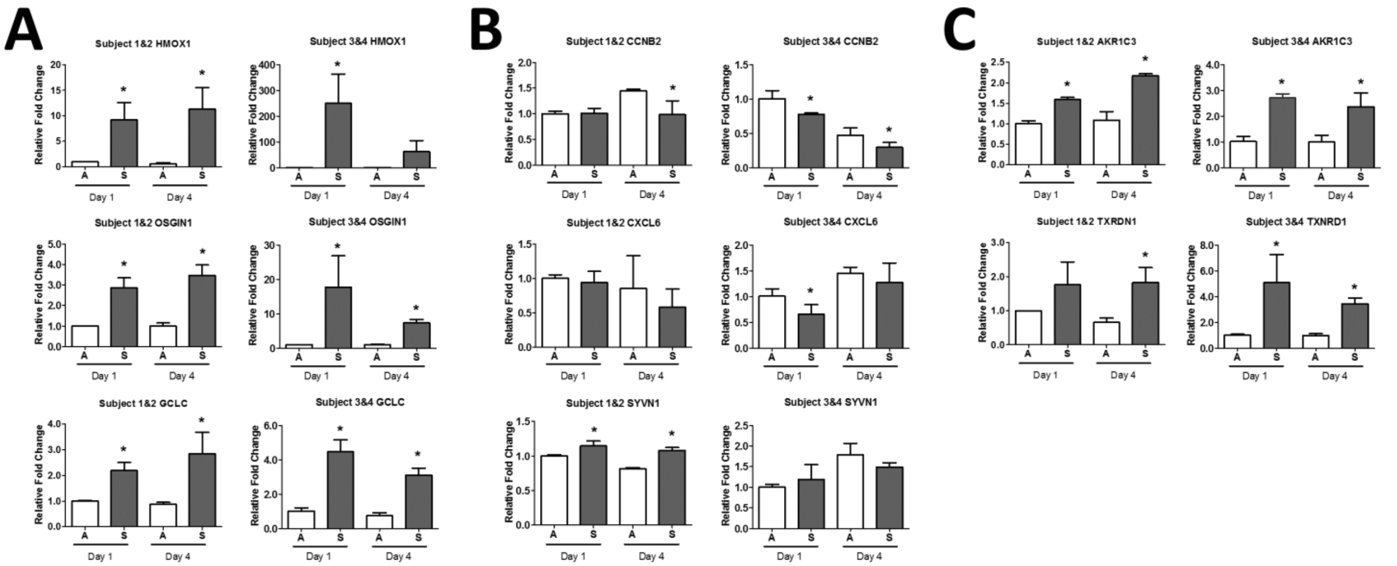

Supplemental Figure 8: Expression of genes that showed differential expression in HBE cells cultured at ALI after smoke treatment were detected in NHBE lines from four independent subjects. Genes shown include those **(A)** identified in Supplemental Figure 6, **(B)** identified in Supplemental Figure 7, and **(C)** associated with smoke-unique H3K27Ac ChIP-Seq peaks. Subjects 1 and 2 were the original HBE cell lines used in the microarray analysis. Subjects 3 and 4 were cultured and treated together at a later time for validation. Subject information is listed in the Supplemental Table 6. Treatment of CS was done as illustrated in Supplemental Figure 5A. A: Air and S: Smoke. * $p<0.05$. Mean+/- SD is shown.

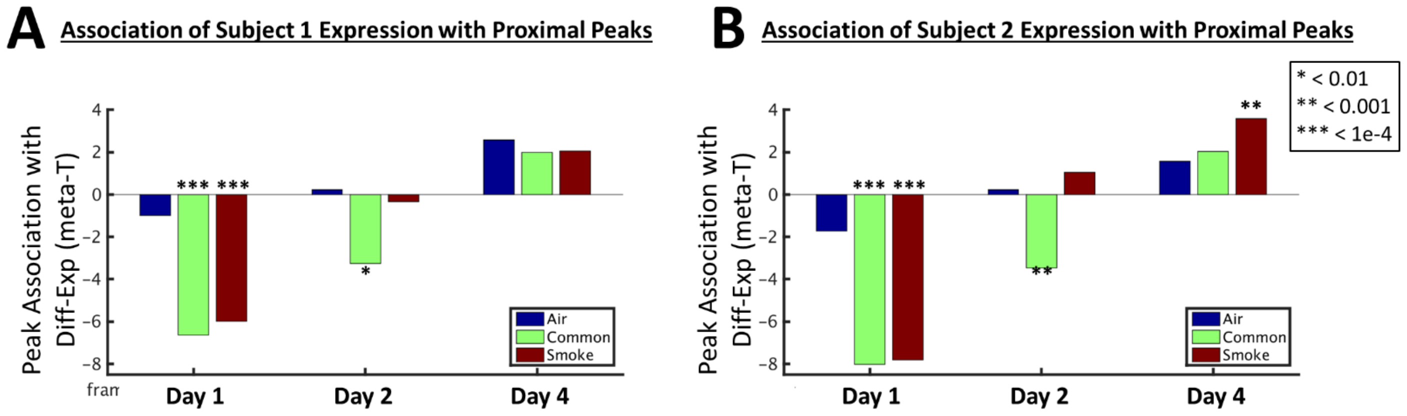

Supplemental Figure 9: The statistical association of genes containing proximal H3K27Ac peaks (identified in subject 1) with the differential-expression levels of genes in smoke compared to air, calculated separately for **(A)** subject 1 expression samples and **(B)** subject 2 expression samples.

Supplemental Tables

Supplemental Table 1: The top smoke unique and air unique peaks identified by MAnorm. Table contains information on the genomic location of the peak, the nearest gene (based on the peak's proximity to the nearest RefSeq annotated transcriptional start site), and the MAnorm significance quantifying how specific a given peak is to either the CS or air condition. Genomic location of peaks is based on hg19 assembly.

Smoke-Unique Proximal Peaks (top 24 based on MA-NORM analysis)		
**Peak Location**	**Nearest Gene**	**-logP value**
chr2:70313333-70314043	PCBP1-AS1	5.73812
chr10:5136767-5137236	AKR1C3	4.06657
chr1:234509226-234509494	COA6	3.30455
chr16:2732192-2732450	KCTD5	3.09993
chr2:74425705-74426133	MTHFD2	3.09993
chr1:234508754-234509148	COA6	2.97345
chr10:101491541-101491833	CUTC	2.9442
chr1:16173629-16173859	SPEN	2.70612
chr9:102860551-102861278	ERP44	2.66531
chr8:95731608-95732033	DPY19L4	2.64075
chr22:41682667-41682966	RANGAP1	2.62629
chr9:132514861-132515276	PTGES	2.51567
chr2:234600602-234600919	UGT1A6	2.51567
chr9:29243-29519	WASH1	2.49326
chr2:183902604-183902948	NCKAP1	2.45517
chr15:81292694-81293197	MESDC1	2.44915
chr1:207226615-207227099	PFKFB2	2.44513
chr1:50888636-50888972	DMRTA2	2.41952
chr11:63933027-63933293	MACROD1	2.41239
chr3:149530387-149530979	RNF13	2.40088
chr5:10249389-10249943	FAM173B	2.37649
chr11:32605304-32605816	EIF3M	2.36704
chr9:139891090-139891513	CLIC3	2.29326
chr3:183966816-183967206	ALG3	2.29326

Air-Unique Proximal Peaks (top 20 based on MA-NORM analysis)		
**Peak Location**	**Nearest Gene**	**-logP value**
chr3:99979338-99979593	TBC1D23	3.91087
chr17:71258048-71258289	CPSF4L	3.54084
chr15:99790923-99791317	LRRC28	3.1488
chr1:26437202-26437507	STK35L2	2.72905
chr4:183837825-183838207	DCTD	2.4748
chr15:80189088-80189382	MTHFS	2.46464
chr2:109237562-109237830	LIMS1	2.38725
chr11:5322795-5323121	OR51B4	2.3749
chr17:49243015-49243658	NME2	2.34986
chr9:5832622-5833093	ERMP1	2.21947
chr4:6783949-6784168	KIAA0232	2.21753
chr9:33024444-33024695	DNAJA1	2.08778
chr19:7059226-7059423	MBD3L3	2.0625
chr1:156737324-156737750	PRCC	2.05089
chr10:102672751-102673005	SLF2	2.01523
chr7:73183901-73184402	CLDN3	1.97657
chr11:64546066-64546293	SF1	1.96542
chr8:6565289-6565721	AGPAT5	1.8576
chr9:139305467-139305777	PMPCA	1.8576
chr14:24610010-24610701	EMC9	1.85122

Supplemental Table 2: (provided as supplemental file) The DAVID functional enrichment analysis results for sets of genes that are proximally (within 1000bp of the transcription start site) located near smoke unique (first tab), common (second tab) or air unique (third tab) peaks, as well as the DAVID functional enrichment analysis results for sets of genes that are distally (within 100kb but farther than 1000bp of the transcription start site) located near smoke unique (fourth tab), common (fifth tab) or air unique (sixth tab) peaks. For each analysis, the term name, percentage of the gene set that is annotated to the term, term size, gene set's fold enrichment in the term, p-value and Benjamini-Hotchberg corrected significance are indicated.

Supplemental Table 3: (provided as supplemental file) The results of differential-expression analysis when comparing CS-exposed and air-exposed samples on day 1, 2, and 4 respectively. In each case the t-statistic value of the test, suggesting higher expression in CS-exposed (positive value) or air-exposed (negative value) samples, p-value, and FDR significance are indicated. Gene symbols are ranked alphabetically.

Supplemental Table 4: (provided as supplemental file) The DAVID functional enrichment analysis on genes that are differentially-expressed ($p<0.05$) with increased (first tab) or decreased (second tab) expression on day 1, increased (third tab) or decreased (fourth tab) expression on day 2, and increased (fifth tab) or decreased (sixth tab) expression on day 4. For each analysis the term name, percentage of the gene set that is annotated to the term, term size, gene set's fold enrichment in the term, p-value and Benjamini-Hotchberg corrected significance are indicated. All GO terms are ranked based on p value from smallest to biggest.

Supplemental Table 5: The 30 genes that showed significant interaction (FDR<0.1) between smoke treatment and exposure duration based on linear mixed models. The gene name, probe ID, p-value and FDR are indicated. Genes are ranked based on p-value from smallest to biggest. The differential expression of these genes in an independent data-set (GSE3717) is also noted in the last two columns. The t-statistic (main number) and FDR (shown in parentheses) of each gene's differential expression with respect to COPD status (yes or no) and smoking status (current or former) is listed. Genes in our analysis that did not have a value in this independent dataset have "NA" values listed.

Gene Symbol	ProbeID	FDR	p-value	DE: COPD/control t-stat (FDR)	DE: current/former smkr t-stat (FDR)
ERCC1	1450356	0.02645744	9.14E-07	4.102165 (0.00164971)	0.311871 (0.851096)
TMF1	2970609	0.02645744	1.80E-06	-1.395130 (0.40096)	1.548519 (0.246924)
EXPH5	7650286	0.02645744	2.37E-06	-0.324613 (0.886911)	3.127302 (0.0093621)
KCTD6	5290193	0.02645744	3.18E-06	0.851681 (0.658474)	-5.404285 (2.71109e-06)
C6orf85	6560328	0.02645744	4.13E-06	NaN (NaN)	NaN (NaN)
IDE	2650523	0.02645744	6.04E-06	0.423231 (0.843467)	6.721301 (5.20505e-09)
C20orf177	770221	0.02645744	9.29E-06	NaN (NaN)	NaN (NaN)
SERTAD1	4290072	0.02645744	9.34E-06	1.459580 (0.373116)	2.473355 (0.0465688)
RAB23	3930047	0.02645744	9.78E-06	-3.151450 (0.0199074)	0.540269 (0.730194)
DPAGT1	6290719	0.02645744	9.99E-06	1.395575 (0.40087)	-3.673853 (0.00187514)
DGCR6	7200274	0.02862513	1.35E-05	-0.917079 (0.628351)	-0.510517 (0.746185)
TMEM68	6060368	0.02862513	1.35E-05	-4.004565 (0.00218099)	-2.831653 (0.0201399)
FAM126B	4880110	0.02862513	1.45E-05	-2.277055 (0.117849)	-6.980596 (1.36109e-09)
LOC100132564	3780767	0.02862513	1.49E-05	NaN (NaN)	NaN (NaN)
RPL41	2680484	0.02910716	1.65E-05	1.189657 (0.49756)	1.863201 (0.151094)
SIL1	6560603	0.02939943	1.79E-05	0.011116 (0.996911)	2.852857 (0.0190915)
REEP3	10593	0.04702189	3.30E-05	2.831539 (0.0406733)	2.791355 (0.0222108)
BAIAP2L1	5700343	0.05000052	4.31E-05	1.889221 (0.217771)	-1.570939 (0.239032)
ST6GAL1	3450706	0.05726716	5.27E-05	3.900210 (0.00293586)	3.262197 (0.00646783)
TNFRSF10B	1710014	0.05892342	6.36E-05	2.821422 (0.0414793)	-5.429646 (2.42137e-06)
LOC442270	5910682	0.06101661	7.07E-05	NaN (NaN)	NaN (NaN)
DYRK1A	2120114	0.06373196	7.67E-05	-0.539101 (0.798975)	-2.259836 (0.0722348)
PIGU	3180554	0.06373196	7.73E-05	-3.223871 (0.0167163)	-4.993015 (1.55763e-05)
YIPF1	1400133	0.06514183	8.29E-05	2.161916 (0.143453)	6.667546 (6.82489e-09)
TST	2230288	0.0760727	0.00011995	-1.358105 (0.41775)	-1.714734 (0.192373)
PLEKHA1	3930541	0.0760727	0.0001291	-1.252205 (0.46835)	0.141520 (0.935707)
CARHSP1	1230348	0.0760727	0.00013616	1.073969 (0.553127)	-4.502822 (0.000108955)
ASAP1	7000072	0.0760727	0.00016012	1.443327 (0.379238)	1.727641 (0.18853)
KIAA1024	1740121	0.0760727	0.00016029	0.416471 (0.847026)	0.936288 (0.516823)
LOC645529	3520279	0.07943146	0.00018205	NaN (NaN)	NaN (NaN)

Supplemental Table 6: Subject information for primary human bronchial cells used in this study.

Subject	Lot	Vendor/ Source	age	gender	smoker	ethnic	Procedure	Related figures and supplemental figures
1	01926	Lifeline	21	Male	Never	Caucasian	Autopsy	Figures 1-5; Supplemental Figures 1-2,4-9
2	00655	Lifeline	5	Female	Never	Caucasian	Autopsy	Figures 4-5 ; Supplemental Figure 5-9
3	UNC24	UNC	18	Male	Never	Black	Autopsy	Supplemental Figure 3, 8
4	UNC7	UNC	16	Female	Never	Caucasian	Autopsy	Supplemental Figure 8

Supplemental Video: (provided as supplemental file) Phase contrast images were taken in fully differentiated HBE cells cultured at air-liquid interface (ALI) in five seconds time frame.

Supplemental References

1 Vaz, M. *et al.* Chronic Cigarette Smoke-Induced Epigenomic Changes Precede Sensitization of Bronchial Epithelial Cells to Single-Step Transformation by KRAS Mutations. *Cancer cell* **32**, 360-376 e366, doi:10.1016/j.ccell.2017.08.006 (2017).
